# The First International Mini-Symposium on Methionine Restriction and Lifespan

**DOI:** 10.3389/fgene.2014.00122

**Published:** 2014-05-09

**Authors:** Gene P. Ables, Holly M. Brown-Borg, Rochelle Buffenstein, Christopher D. Church, Amany K. Elshorbagy, Vadim N. Gladyshev, Tsang-hai Huang, Richard A. Miller, James R. Mitchell, John P. Richie, Blanka Rogina, Martha H. Stipanuk, David S. Orentreich, Norman Orentreich

**Affiliations:** ^1^Orentreich Foundation for the Advancement of Science, Cold SpringNY, USA; ^2^School of Medicine & Health Sciences, University of North Dakota, Grand ForksND, USA; ^3^University of Texas Health Science Center at San Antonio, San AntonioTX, USA; ^4^Harwell Science and Innovation CampusOxfordshire, UK; ^5^Department of Pharmacology, University of OxfordOxford, UK; ^6^Department of Physiology, University of AlexandriaAlexandria, Egypt; ^7^Department of Medicine, Brigham and Women's Hospital, Harvard Medical School, Harvard University, BostonMA, USA; ^8^Institute of Physical Education, Health and Leisure Studies, National Cheng Kung UniversityTainan, Taiwan; ^9^Geriatrics Center and Institute of Gerontology, University of Michigan, Ann ArborMI, USA; ^10^Department of Genetics and Complex Diseases, Harvard School of Public Health, Harvard UniversityBoston, MA, USA; ^11^Public Health Sciences, College of Medicine, Penn State UniversityHershey, PA, USA; ^12^University of Connecticut Health Center, FarmingtonCT, USA; ^13^Division of Nutritional Sciences, Cornell UniversityIthaca, NY, USA

**Keywords:** methionine restriction, lifespan, aging and longevity, animal models

## Abstract

It has been 20 years since the Orentreich Foundation for the Advancement of Science, under the leadership Dr. Norman Orentreich, first reported that low methionine (Met) ingestion by rats extends lifespan (Orentreich et al., 1993). Since then, several studies have replicated the effects of dietary methionine restricted (MR) in delaying age-related diseases (Richie et al., 1994; Miller et al., 2005; Ables et al., 2012; Sanchez-Roman and Barja, 2013). We report the abstracts from the First International Mini-Symposium on Methionine Restriction and Lifespan held in Tarrytown, NY, September 2013. The goals were (1) to gather researchers with an interest in MR and lifespan, (2) to exchange knowledge, (3) to generate ideas for future investigations, and (4) to strengthen relationships within this community. The presentations highlighted the importance of research on cysteine, growth hormone (GH), and ATF4 in the paradigm of aging. In addition, the effects of dietary restriction or MR in the kidneys, liver, bones, and the adipose tissue were discussed. The symposium also emphasized the value of other species, e.g., the naked mole rat, Brandt's bat, and *Drosophila*, in aging research. Overall, the symposium consolidated scientists with similar research interests and provided opportunities to conduct future collaborative studies (**Figure 3**).

Amany K. Elshorbagy (University of Oxford, UK, and University of Alexandria, Egypt) presented “Sulfur amino acids and body composition.” Emerging evidence from knockout studies points to involvement of sulfur amino acids in regulation of body composition. Homozygous deletion of cystathionine beta-synthase, which initiates cysteine synthesis from homocysteine, reduces body fat in mice. Mice with a genetic defect in glutathione synthesis have increased energy expenditure and resist obesity. A similar phenotype is observed in rats fed methionine restricted (MR) diets. Common to these models are decreased cysteine synthesis and/or plasma total cysteine (tCys) and profound hepatic suppression of the key lipogenic enzyme stearoyl-coenzyme A desaturase-1 (SCD1). Supplementation of cysteine, but not taurine, reverses the MR-induced SCD1 suppression and restores fat gain in rats. In mice, high cystine intake lowers energy expenditure and up-regulates lipogenic and diabetogenic enzymes. In humans, plasma tCys correlates with estimated SCD activity and fat mass. Among upstream compounds, only *S*-adenosylmethionine shows similarly strong associations with fat mass. In adipocytes, cysteine inhibits lipolysis. Pilot drug studies in mice show that lowering cysteine concentrations can decrease the fat mass gain caused by a high-fat diet (HFD; **Figure 1**). Experimental and epidemiologic data suggest a role for sulfur amino acids in regulation of energy metabolism, in part via an influence on SCD1.

**FIGURE 1:**
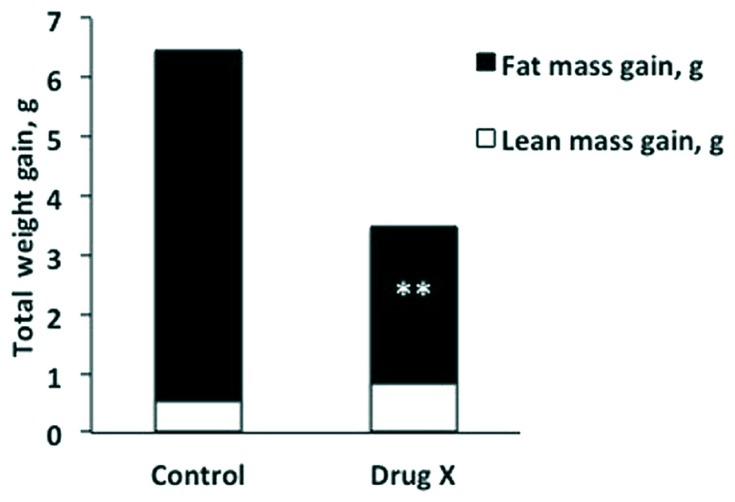
**Total weight gain broken down into lean mass gain (white) and fat mass gain (black) in mice after 2 weeks on a high-fat diet, with or without a drug that lowers plasma total cysteine (Drug X).** The cysteine lowering drug halved fat mass gain without compromising lean mass. **P < 0.001.

Martha H. Stipanuk (Cornell University, USA) presented “Regulation of cysteine dioxygenase (CDO) in response to sulfur amino acid intake: is minimizing H_2_S production the goal?” The sulfur of sulfur-containing amino acids eventually makes its way to inorganic sulfur or taurine as metabolic end-products that can be excreted in the urine. Cysteine is metabolized both by a direct oxidation pathway, in which the thiol group of cysteine is oxygenated prior to further catabolism to either sulfate or taurine, and by desulfhydration-oxidation pathways, in which the reduced sulfur is released from the carbon chain as H_2_S/HS^-^ or sulfane sulfur prior to its further step-wise oxidation to sulfate. Cysteine flux through these pathways is a function primarily of the activity of CDO, which initiates the flux of cysteine through the direct oxidative pathway, and of cysteine concentration, which is a large determinant of flux through desulfhydration pathways as well as of CDO abundance and activity. CDO is robustly regulated in response to cysteine availability, suggesting that control of cellular cysteine levels is critical. Studies with *Cdo1* knockout mice suggest that upregulation of CDO in response to cysteine availability serves to prevent the production of excess levels of H_2_S/HS^-^ when sulfur amino acid intake is high. Mice lacking CDO metabolize excess cysteine by desulfhydration pathways, leading to high exposure of tissues to endogenously produced H_2_S/HS^-^. These mice exhibit postnatal growth deficits and connective tissue pathologies, but they also exhibit a lean phenotype, being resistant to diet-induced obesity/insulin insensitivity. Future studies will be aimed at defining the beneficial and harmful effects of elevated H_2_S/HS^-^ exposure as well as effects of the lack of hypotaurine/taurine.

Holly M. Brown-Borg (University of North Dakota, USA) presented “Growth hormone (GH) and methionine (Met): interactions in aging and longevity.” Endocrine hormones impact aging and aging processes in multiple ways. Circulating GH affects not only somatic growth but also drives aspects of metabolism. We have previously shown that GH modulates Met metabolism in GH-deficient mice. Restricting Met in rodent diets has been shown to lower insulin-like growth factor-1 (IGF-1) and extend lifespan. Our current studies focus on delineating the relationships between dietary methionine, plasma GH status, and factors involved in stress resistance. Our working hypothesis is that GH is involved in the regulation of thiol metabolism that, in turn, affects an organism’s resistance to stressors and ultimately impacts lifespan. Ames dwarf, GH transgenic, and respective wild type mice (*n* = 40–60/group) were subjected to dietary MR or enrichment. Following eight weeks on the Met diets, components of the glutathione and Met metabolic pathways were examined. Plasma IGF-1 levels declined with decreasing dietary Met content. Gene expression of Met conserving and catabolizing enzymes was differentially affected by dietary Met level. Underlying GH status also influenced the metabolic responses to altered dietary Met. Lifespan studies using Ames dwarf and GH transgenic animals subjected to diets restricted or enriched with Met are currently underway. At this point, wild type mice respond to the Met diets as expected, living longer on low Met vs. higher levels (*p* < 0.0001); however, dwarf mice do not appear to respond to altered Met in the diet at this point in the study. The GH transgenic animals live much longer on MR diets when compared to published lifespans for these animals yet do not outlive their wild type counterparts on either of the diets tested (*p* < 0.0001). The results to date suggest that the level of circulating GH interacts with dietary Met and alters metabolism and lifespan in mice.

James R. Mitchell (Harvard School of Public Health, USA) presented “Contribution of essential amino acid restriction to the benefits of short-term dietary restriction (DR) in mice.” DR, defined as reduced food intake without malnutrition, can increase lifespan, metabolic fitness, and/or stress resistance when applied for long periods of time in experimental organisms. However, short-term DR lasting only one week can precondition against clinically relevant stressors, such as ischemia reperfusion injury seen as a frequent complication of cardiovascular surgery. Previously, we showed that removal of protein or specific essential amino acids (tryptophan, leucine, or Met) could precondition against surgical stress in a mouse model of renal ischemia. We also demonstrated a genetic requirement for the amino acid deprivation sensing kinase, GCN2. Here, we found that calorie restriction and essential amino acid restriction contributed additively to the benefits of DR against surgical stress. Adding back essential amino acids abrogated the protection afforded by protein restriction independent of their calorie content. An increase in AMPK activity and decrease in mTORC1 activity correlated with functional benefits. These findings have translational implications for evidence-based dietary recommendations before elective surgery and other forms of acute stress in which ischemia reperfusion injury can play a role.

Gene Ables (Orentreich Foundation for the Advancement of Science, USA) presented “Metabolic effects of dietary MR in mice.” Dietary MR in rodents extended lifespan and protected them from developing obesity and diabetes. Since the recruitment of adipose tissue macrophages is implicated in the metabolic syndrome, we asked whether MR reduces its accumulation. To test this hypothesis, lean and diet-induced obese (DIO) mice were fed isocaloric HFD containing either 0.86% (CF) or 0.12% (MR) Met. MR mice on HFD had lower body weight despite increased food intake. These mice were more insulin sensitive with reduced hepatic triglyceride accumulation. These mice had higher plasma levels of adiponectin and fibroblast growth factor 21 (FGF21), while leptin, serum amyloid A (SAA) and IGF-1 levels were reduced. The hepatic genes of the MR mice showed the down-regulation of *Scd1*, while *Pparg*, *Atgl*, *Cd36*, *Jak2*, and *Fgf21* were upregulated. The smaller perigonadal adipose tissue (PGAT) in MR mice showed lower gene expressions for *Emr1*, *Cd68*, *Ccr2*, *Itgam*, and *Tnfa* and a decrease in F4/80 protein staining. The DIO-MR mice exhibited weight loss with improved glucose metabolism. PGAT genes showed decreased *Ccl2* expression, while *Atgl* was increased in the DIO-MR mice. Staining for F4/80 in the PGAT showed decreased expression in the DIO-MR mice, which also had smaller adipocyte size. Overall, our data suggest that MR protects mice from obesity and diabetes with concomitant reduction of adipose tissue macrophage accumulation.

Tsang-Hai Huang (National Cheng Kung University, Taiwan) presented “Effects of MR diet and endurance exercise (EXE) training on bones in male growing rats.” MR diets as well as EXE have been mentioned to be associated with lower bone mass. Whether this reduced bone mass represented compromise in bone quality needs to be further clarified. The purpose of this study was to investigate the effects MR and EXE on bone quality. In experiment 1, young male SD rats were assigned to diets containing 0.86, 0.52, and 0.17% Met for 10 days. In a second experiment, animals were assigned to six groups fed with the same Met diets combined with or without EXE for 8 weeks. In both experiments, the 0.17% Met fed groups showed significant reduction in body weight, longitudinal growth, and trabecular bone volume. Significant down-regulation of osteocalcin was shown in the 0.17% Met combined EXE group. Serum bone resorption markers were significantly down-regulated due to 0.17% Met feeding and/or EXE in both experiments. Dynamic histology analyses revealed a significant reduction in cortical and/or spongy bone formation rates in 0.17% Met fed animals. And, EXE revealed significant down-regulation in osteoclast density. Both MR and EXE caused lower bone mineral content (BMC) measurements, but total BMD of 0.17% Met fed rats was significantly higher in experiment 1 and lower in 0.17% Met plus EXE group of experiment 2. Femora of 0.17% Met fed rats revealed significantly lower extrinsic (whole bone level) mechanical properties (e.g., bending load and energy), but no difference (Experiment 1) and stronger (Experiment 2) in intrinsic (material level) mechanical properties (e.g., bending stress, toughness, and elastic modulus). Moreover, EXE provided an enhancement effect in tissue-level properties (e.g., yield stress and toughness) for the 0.86% Met fed animals. Conclusion: MR diets and EXE showed down-regulated effects on bone/energy metabolic indices, bone mass, and/or whole bone strength without further compromising intrinsic bone mechanical properties. The effects of MR and/or EXE in aging bone are worthy of further study.

Vadim N. Gladyshev (Brigham and Women’s Hospital, Harvard University Medical School, USA) presented “Understanding control of lifespan through genome analyses and methionine status.” Understanding the mechanisms that control lifespan is among the most challenging biological problems. Many complex human diseases are associated with aging, which is both the most significant risk factor and the process that drives the development of these diseases. The aging process can be regulated during evolution. For instance, mammals are characterized by >100-fold difference in lifespan, which can both increase and decrease during evolution. We employed this diversity in mammalian lifespan and the associated life-history traits to shed light on mechanisms that regulate species lifespan. For this, we utilized methods of comparative genomics to examine genomes of short- and long-lived species and carry out analysis of lifespan across a panel of mammals. We sequenced the genomes of mammals with exceptional lifespans, including the naked mole rat and the Brandt’s bat, and identified candidate genes that may contribute to their longevity. In addition, one factor that emerged as relevant to the control of lifespan is Met availability. Indeed, reduced Met intake can extend lifespan in rodents by mimicking DR, but whether this regimen represents a general strategy for regulating aging has been controversial. We found that MR could extend lifespan of model organisms in the area of aging, but this effect was limited to specific dietary conditions. These studies provide insights into the roles of Met in aging and suggest a strategy for lifespan extension by MR. It is our hope that a better understanding of molecular mechanisms of mammalian lifespan control will lead to a better understanding of human diseases of aging.

John P. Richie, Jr. (Penn State University College of Medicine, USA) presented “Translational studies on dietary MR.” Previous findings in rodent models that dietary MR increases maximum lifespan and reduces the development of aging-related impairments suggest that MR may have important implications as a preventive or therapeutic strategy in humans. However, to date, there have been few studies aimed at translating these pre-clinical findings to the clinic. To this end, we conducted a short-term controlled cross-over feeding study of MR in healthy adults. This study consisted of two isocaloric diet groups (control and 86% MR). Our objectives were to determine the feasibility of feeding an MR diet and to assess the effects of MR on relevant blood biomarkers. The study was conducted with 12 healthy adults and consisted of two 3-week experimental feeding periods with a 2-week washout (**Figure 2**). The MR diet was well-tolerated by all subjects with no negative side-effects reported. Decreases in plasma levels of Met (22%) and cysteine (15%) were observed in the MR group after 3 weeks. MR significantly decreased plasma total cholesterol (15%), LDL (23%), and uric acid (25%), but had no effects on leptin, adiponectin, IGF-1, or glutathione. Altogether, these findings demonstrate the feasibility of a MR diet in humans and indicate that MR has significant short-term effects on blood lipids similar to those observed in laboratory animal models. In addition, the lack of effects on blood adipokines and glutathione are consistent with more recent laboratory findings that indicate that restrictions in both Met and Cys may be required for the full range of beneficial effects on adipokines and longevity.

**FIGURE 2:**
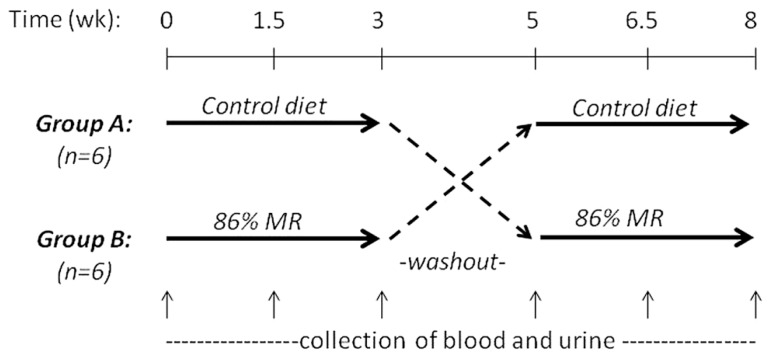
**MR randomized controlled crossover feeding study design**.

**FIGURE 3:**
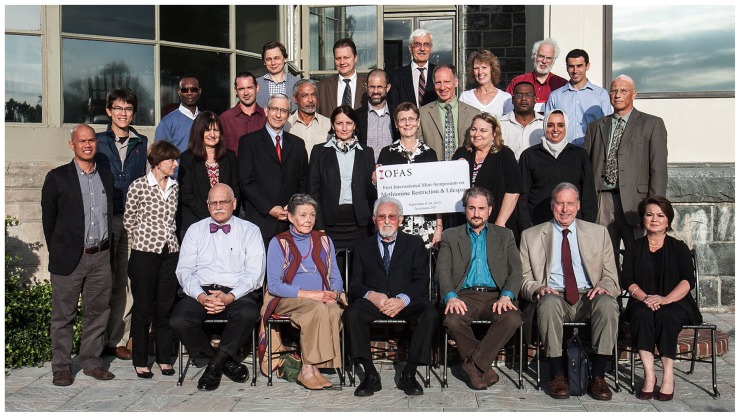
**Assembled participants of the First International Mini-Symposium on Methionine Restriction and Lifespan (Tarrytown, NY, USA, September 8–10, 2013).** Sitting in the front row (L–R): Jay Zimmerman (Moderator), Nancy P. Durr, Norman Orentreich, David S. Orentreich, George Roth (Keynote Speaker), Bernardita Calinao. Standing 1st row (L–R): Gene P. Ables, Ines Augie, Virginia Malloy, Herb Burack, Blanka Rogina, Martha H. Stipanuk, Rochelle Buffenstein, Amany K. Elshorbagy, John P. Richie. Standing 2nd row (L–R): Tsang-hai Huang, Amadou Ouattara, Jason Plummer, Franz Perrodin, James R. Mitchell, Joel Brind, Dwight Mattocks, Christopher D. Church. Last row (L–R): Vadim N. Gladyshev, Boris Krasnikov, Arthur Cooper (Moderator), Holly M. Brown-Borg, and Richard A. Miller.

Richard A. Miller (University of Michigan, USA) presented “Drugs, diets, genes that extend mouse lifespan: any common pathways?” The last 20 years have produced convincing evidence that maximum lifespan in rodents can be increased by at least two diets, mutation of at least half a dozen genes, and at least one drug, rapamycin. Evidence that the longevity effect reflects slower aging, rather than, say, anti-cancer effects on their own, is very strong for caloric restriction and fairly strong for MR, several of the mutations that block GH and IGF-1 signals, and rapamycin. These new research tools allow initial studies of an interesting question: are there “common pathways” that are altered in parallel in each of these nominally different approaches for slowing the aging process? This presentation focused on two candidate mechanisms: (a) induction of enzymes that lead to detoxification of xenobiotic and endogenously generated metabolites and (b) activation of ATF4, a protein that senses blocks to translation. The talk also presented data on four newer approaches to lifespan extension in mice: (a) the crowded litter (CL) model, based on transient early life nutritional deprivation; (b) acarbose, a drug that slows digestion of starches to sugars and extends lifespan, principally in males; (c) nordihydroguaiaretic acid, NDGA, an anti-inflammatory agent that extends male lifespan; and (d) MIF-KO, a mutation that lowers levels of Migration Inhibitory Factor, a pro-inflammatory cytokine with links to insulin secretion and response. Work now in progress has shown that liver from MR mice has elevations in ATF4 protein and multiple ATF4 target mRNAs, suggesting that further study of the role of translation control and stress resistance in the restricted mice may give clues to connections between diet and late-life disease.

Rochelle Buffenstein (University of Texas Health Science Center, USA) presented “Stress-enhanced proteostasis in long-lived naked mole-rats.” Improved cellular stress resistance is commonly associated with extended species longevity, although the mechanisms involved remain elusive. It is likely that these traits are linked to dietary protein content and the efficient removal of damaged proteins. Naked mole-rats are strictly herbivorous and naturally have a low met diet. This lifestyle may contribute to their cancer-free good health for 75% of their preternaturally long, 32-year lifespan, despite high levels of oxidative stress present from an early age. Clearly both genomic and proteomic integrity are well maintained in this mouse-sized rodent. Unlike mice that show a pronounced decline in the removal of damaged proteins by the proteasome both with age and in response to stress, mole-rats maintain four-fold higher proteasome activities than do mice over at least 75% of their lifespan. Moreover, mole-rat proteasomes are also resistant to inhibition by proteasome-specific competitive inhibitors (e.g., bortezomib) as well as oxidative stressors such as 4-hydroxynonenal and dietary toxins such as heavy metals. Maintaining proteasome activity and preventing their inhibition by damaging agents, even when subjected to oxidative insults and dietary toxins, likely contributes to their well maintained protein homeostasis and their concomitant ability to stave off the progressive decline and other vagaries associated with the aging process. We observed that a cytosolic factor protects the proteasome from inhibition and also modulates its activity. In addition, we present evidence that this cytosolic protective factor is transferrable and capable of protecting human, yeast, and mouse proteasomes, promoting resistance to inhibition by toxins and proteasome specific inhibitors and also augmenting their activity even in the absence of inhibitory agents. Thus, protein assembly that is capable of protecting proteasomes from an evolutionarily diverse suite of organisms likely plays a pivotal role in stress resistance and species longevity and may also contribute to the ability of naked mole-rats to successfully defy the aging process.

Blanka Rogina (University of Connecticut Health Center, USA) presented “*Indy* mutations maintain fly health and homeostasis.” *Indy* (*I’m not dead yet*) encodes the fly homolog of a mammalian transporter of the Krebs cycle intermediates. Reduced *Indy* gene activity has beneficial effects on energy balance in mice, worms, and flies, and on worm and fly longevity. In flies, longevity extension is not associated with negative effects on fertility, mobility, or metabolic rate. We and others show that *Indy* mutations extend longevity by mechanism similar to calorie restriction. Some of the hallmarks of these changes are altered intermediate nutrient metabolism and increased mitochondrial biogenesis. These changes have been found in fly heads, thoraces, and the midguts. The observed changes in midgut energy metabolism, specifically decreased production of free radicals, results in preservation of intestinal stem cell (ISC) homeostasis, which is characterized by a decrease in age-associated accumulation of ISCs. Our studies show a direct connection between changes in energy metabolism, caused by the *Indy* mutation, and preservation of ISC homeostasis and midgut integrity. The data suggest that *Indy* mutations preserve homeostasis in tissues that contribute to extended health and longevity.

Christopher D. Church (Harwell Science and Innovation Campus, Oxfordshire, UK) presented “The fat mass and obesity associated gene (FTO) regulates body composition and energy homeostasis.” Genome-wide association studies have identified single nucleotide polymorphisms within the human FTO that display strong association with obesity and type 2 diabetes. Individuals homozygous for the at-risk allele weigh, on average, ~3 kg more than individuals with the low-risk allele and display increased food intake, including a preference for fat and protein. Bioinformatic and *in vitro* analyses show that FTO codes for a AlkB-like, Fe(II)- and 2-oxoglutarate-dependent nucleic acid demethylase that catalyzes the demethylation of 3-methylthymine, *N*6-methyladenosine, and 3-methyluracil in single-stranded DNA and RNA. Rodent studies have demonstrated a role for FTO in energy homeostasis and body composition. Mice over-expressing *Fto* are obese, hyperphagic, and exhibit normal energy expenditure. Conversely, the constitutive germline loss of *Fto* results in high perinatal lethality and a reduction in body length, fat mass, and lean mass. More recently, we have shown that inactivation of *Fto* in adult mice leads to reduced body weight, primarily due to a loss of lean mass, and lower RER. Furthermore, selective inactivation of *Fto* in the hypothalamus, a key site for the nutritional regulation of *Fto* expression, results in reduced food intake and body weight gain. Additionally, *in vitro* experiments have identified that *Fto* expression is dynamically regulated by essential amino acid availability, including glutamine, cysteine, and Met. The altered substrate utilization and sensing of dietary macronutrients highlight a critical role for FTO in regulating peripheral metabolism and provide mechanistic insights into how FTO contributes to obesity.

## Conflict of Interest Statement

The authors declare that the research was conducted in the absence of any commercial or financial relationships that could be construed as a potential conflict of interest.

## References

[B1] Orentreich N. Matias J. R. DeFelice A. Zimmerman J. A. (1993). Low methionine ingestion by rats extends life span. *J. Nutr.* 123 269–274 8429371 10.1093/jn/123.2.269

[B2] Ables G. P. Perrone C. E. Orentreich D. Orentreich N. (2012). Methionine-restricted C57BL/6J mice are resistant to diet-induced obesity and insulin resistance but have low bone density. *PLoS ONE* 7:e51357 10.1371/journal.pone.0051357 PMC3518083 23236485

[B3] Miller R. A. Buehner G. Chang Y. Harper J. M. Sigler R. Smith-Wheelock M. (2005). Methionine-deficient diet extends mouse lifespan, slows immune and lens aging, alters glucose, T4, IGF-I and insulin levels, and increases hepatocyte MIF levels and stress resistance. *Aging Cell* 4 119–125 10.1111/j.1474-9726.2005.00152.x 15924568 PMC7159399

[B4] Richie J. P. Jr. Leutzinger Y. Parthasarathy S. Malloy V. Orentreich N. Zimmerman J. A. (1994). Methionine restriction increases blood glutathione and longevity in F344 rats. *FASEB J*. 8 1302–1307 8001743 10.1096/fasebj.8.15.8001743

[B5] Sanchez-Roman I. Barja G. (2013). Regulation of longevity and oxidative stress by nutritional interventions: role of methionine restriction. *Exp. Gerontol*. 48 1030–1042 10.1016/j.exger.2013.02.021 23454735

